# Patterns of care and survival for patients aged under 40 years with bone sarcoma in Britain, 1980–1994

**DOI:** 10.1038/sj.bjc.6602885

**Published:** 2005-11-29

**Authors:** C A Stiller, S J Passmore, M E Kroll, P A Brownbill, J C Wallis, A W Craft

**Affiliations:** 1 Childhood Cancer Research Group, University of Oxford, 57 Woodstock Road, Oxford OX2 6HJ, UK; 2 Sir James Spence Institute of Child Health, Royal Victoria Infirmary, Newcastle upon Tyne NE1 4LP, UK

**Keywords:** osteosarcoma, Ewing's sarcoma, survival, patterns of care

## Abstract

The purpose of the study was to calculate population-based survival rates for osteosarcoma (OS) and Ewing's sarcoma (ES) in Great Britain during 1980–1994, determine proportions of patients treated at specialist centres or entered in national and international clinical trials, and investigate effects of these factors on survival. Data on a population-based series of 1349 patients with OS and 849 with ES were compiled from regional and national cancer registries, UK Children's Cancer Study Group, regional bone tumour registries and clinical trials. Follow-up was through population registers. Survival was analysed by actuarial analysis with log-rank tests and by Cox's proportional hazards analysis. Five-year survival rates during 1980–1984, 1985–1989 and 1990–1994 were 42% (95% CI: 37, 46), 54% (95% CI: 50, 59) and 53% (95% CI: 48, 57), respectively, for OS and 31% (95% CI: 26, 37), 46% (95% CI: 40, 51) and 51% (95% CI: 45, 57) for ES. Proportions of patients treated at a supraregional bone tumour centre or a paediatric oncology centre in the three quinquennia were 36, 56 and 67% for OS and 41, 60 and 69% for ES. In 1983–1992, 48% of OS patients were entered in a national trial; for ES, 27% were entered in 1980–1986 and 54% in 1987–1994. Survival was similar for trial and nontrial patients with OS. For ES, trial patients had consistently higher 5-year survival than nontrial patients: 1980–1986, 42 *vs* 30%; 1987–1992, 59 *vs* 42%; 1993–1994, 54 *vs* 43%. During 1985–1994, patients with OS or ES whose main treatment centre was a nonteaching hospital had lower survival rates. In multivariate analyses of patients diagnosed during 1985–1994 that also included age, sex, primary site, surgical treatment centre, the results relating to main treatment centre for both OS and ES retained significance but the survival advantage of trial entry for ES became nonsignificant. For both OS and ES diagnosed since 1985, patients whose main treatment centre was a nonspecialist hospital had a lower survival rate.

Among persons aged under 40 years, osteosarcoma (OS) and Ewing's sarcoma (ES) are the most frequently occurring malignant bone tumours (Draper, 1985; Hartley *et al*, 1991). Some cases of chondrosarcoma also occur, although this is usually a low-grade tumour often curable by surgery alone.

Survival rates for children aged under 15 years in Great Britain have improved substantially (Stiller, 1994). For OS, two-year actuarial survival increased from 45% in 1980–82 to 68% in 1989–91, while for ES there was an improvement from 55 to 85% over the same period.

Adults with OS have a worse prognosis than children (Hartley *et al*, 1992). In a national study of cancer survival trends in England and Wales, five-year relative survival rates for adults with bone cancer diagnosed during 1971–1975, 1976–1980, 1981–1985 and 1986–1990 were 29, 36, 40 and 51%, respectively (Coleman *et al*, 1999). These results were for all patients aged 15 years and over, however, and did not distinguish between histological types.

In England and Wales, age-standardised mortality from all malignant bone tumours (ICD 170) at age 10–19 years (based on a uniform population) fell from 8.9 per million in 1981–1984 to 5.7 in 1989–1992, a decrease of 36%, whereas at age 20–44 years it fell from 2.9 per million in 1981–84 to 2.7 in 1989–92, a decrease of only 6%. As there is no evidence for any substantial change in incidence, and allowing for the interval between diagnosis and death (among children, most deaths from bone cancer occur at 1–2 years after diagnosis though there is substantial further mortality for some years after), these results strongly suggest that the improvements in survival from bone sarcoma in children which took place during the 1980s extended to patients diagnosed at up to age 17 or 18 years. For older patients, however, there is little evidence from mortality data that there has been any improvement in survival.

There was a substantial improvement in the survival of children with OS in Britain between the late 1970s and mid 1980s (Stiller and Bunch, 1990) and this was concentrated among children treated at paediatric oncology centres, who during 1981–1984 had a significantly higher survival rate than those treated elsewhere (Stiller, 1988). For children with ES, there was little change in national survival rates over the same period (Stiller and Bunch, 1990), but throughout 1977–1984 there was a significantly higher survival rate among children treated at paediatric oncology centres (Stiller, 1988).

The only published study of patterns of care and survival from bone tumours in adults concerned patients with OS from the South Thames Regions of England registered during 1963–82, almost entirely before the more recent increase in survival of young patients associated with more effective chemotherapy (Gill *et al*, 1988). The effect of entry to trials on survival from bone sarcoma has not previously been assessed.

The objectives of the present study were to calculate population-based survival rates for OS and ES among patients aged under 40 years; to determine why the marked decrease in bone cancer mortality among the population aged under 20 years has not been seen in the 20–39 year age group; to determine the proportion of patients treated at specialist centres or entered in clinical trials and to investigate the effects of these factors on survival.

## PATIENTS AND METHODS

Virtually all patients aged under 15 years at diagnosis were already included in the National Registry of Childhood Tumours (NRCT) (Stiller *et al*, 1995). For the age group 15–39 years, the principal sources were the regional cancer registries in England and the national cancer registries of Scotland and Wales. Many young people with bone sarcomas are treated by members of the UKCCSG and included in the UKCCSG Register; for those aged 15 years and over, the UKCCSG Register was an independent source of ascertainment; UKCCSG patients below this age were already in the NRCT. Specialist bone tumour registers in several regions and the Birmingham Bone Tumour Service supplied lists of eligible cases. Lists of patients entered in national and international trials for OS and ES were provided by the Medical Research Council (MRC) Cancer Trials Office and the UKCCSG, respectively, and linked with registration records.

Incident cases of bone cancer during the study period were classified on the basis of the best available information on the diagnosis for each case.

There were 2843 patients with a malignant bone tumour diagnosed as a first cancer before age 40 years during 1980–1994. The 32 patients who had a bone cancer as a second or later malignancy are not considered here.

Osteosarcoma was the most common subgroup, accounting for 1349 cases (47%). Incidence was highest at age 10–19 years. Overall, 59% of OSs were diagnosed in males. The proportion of males was highest at age 15–24 years (64%), while among children aged under 10 years there was a slight female excess (55%). Osteosarcoma is predominantly a tumour of the long bones of the limbs. In this series, 75% of cases with known primary site arose in the legs and 12% in the arms. Only 11% of primaries were in the axial skeleton. The proportion of axial primaries was higher at age 25 years and over (28%).

Ewing's sarcoma, including peripheral primitive neuroectodermal tumour (pPNET) of bone, was the second most common subgroup, with 849 (30%) cases. In the first 10 years of life it was the most frequent type of bone cancer. Incidence reached its peak at age 10–14 years and declined sharply throughout early adulthood. The overall male excess (58%) was similar to that for OS, and it was less pronounced (53%) among children aged under 15 years. The most frequent primary sites for ES were the leg (35% of tumours of known primary site) and the pelvis (26%). Pelvic primaries were extremely rare before the age of 5 years but otherwise there was little variation in primary site with age.

Chondrosarcoma accounted for 307 (11%) cases, other specified tumours for 245 (8%) and unspecified tumours for 93 (3%).

The present analyses of patterns of care and survival are restricted to OS and ES.

Main (nonsurgical) treatment centre for clinical trial patients was defined as the hospital from which the patient was enrolled in the trial. For nontrial patients, it was defined as the highest level hospital (in the classification below) at which there was evidence from any data source that the patient received nonsurgical treatment within 2 months of diagnosis. Treatment centres were classified as follows:

BTS: the two supra-regional Bone Tumour Services in London and Birmingham; UKCCSG: the 20 paediatric oncology centres affiliated to the UK Children's Cancer Study Group (from 1990, some London BTS patients were registered with the UKCCSG but for all the analyses presented here they have been counted as BTS); Other teaching hospitals: the remaining 26 hospitals in geographical proximity to and attached to medical schools; Nonteaching hospitals: the remaining 82 hospitals treating study patients.

Surgical centres were identified from registrations and grouped as BTS and all other hospitals.

All study patients in the NRCT for whom a death certificate has not been received are flagged in the National Health Service Central Registers (NHSCR). Death certificates for persons dying of neoplasms at age 15–19 years are also available from the NRCT. Study patients aged 15–39 years at diagnosis were submitted for flagging at NHSCR. NHSCR provided death certificates for flagged patients who had died, together with notifications of emigration resulting in loss to follow-up. All flagged patients not known to have died or emigrated were assumed to be alive on 31st January 2003. Follow-up information was available for 96% (1297) of patients with OS and 98% (831) of those with ES.

Survival rates were calculated by the Kaplan–Meier method and univariate analyses performed using the log-rank test (Peto *et al*, 1977). Multivariate analyses of survival were carried out using Cox's proportional hazards analysis (Cox, 1972). All survival analyses were carried out using the statistical software package Stata, Version 7 (StatCorp, College Station, TX, USA).

## RESULTS

### Referral patterns

Table 1 shows the numbers of patients with OS and Ewing's sarcoma classified by main treatment centre. Referral to BTS increased in successive periods, reaching 38% of patients for whom this information was known for OS and 30% for ES by 1990–1994. Between 1980–1984 and 1990–1994, the proportions referred to nonteaching hospitals fell from 24 to 5% for OS and from 20 to 7% for ES. Referral to hospitals with fewer than two new study patients per year also fell, with the decrease being more marked for OS (from 36 to 10%) than for ES (from 31 to 19%). The proportion of patients at nonteaching centres increased with age for both diagnostic groups.

Information on surgical centre was available from 1982 onwards. Overall, 53% (612/1151) of patients with OS were referred to a BTS for surgery. The proportion so referred increased from 35% in 1982–1984 to 54% in 1985–1989 and 65% in 1990–1994. Referral rates by age were 55% at 0–9 years, 65% at 10–14, 52% at 15–19 and 45% at 20–39 years. For ES, local therapy has often been by radiotherapy alone for axial primaries (Craft *et al*, 1998) and also for many limb primaries until the mid 1980s (Craft *et al*, 1997). Surgical centre was therefore only analysed for patients with limb primaries diagnosed during 1985–1994. Among this group, 51% (139 out of 274) were referred to a BTS for surgery. The referral rate varied little with age (results not shown).

### Entry to national clinical trials

During 1983–1986, there was one OS trial for which patients aged under 40 years with operable tumours were eligible (Bramwell *et al*, 1992), including those with metastases at diagnosis (Bramwell *et al*, 1997). While intended for patients with limb primaries, this trial also included a few with axial tumours. During 1987–1992, two trials were open simultaneously. The first was for patients with localised limb primaries (Souhami *et al*, 1997) and the second was for patients with metastatic disease or axial tumours (Voûte *et al*, 1999). Entry rates overall were similar in 1983–1986 (47%) and 1987–1992 (50%). They were higher for children and adolescents aged under 20 years (55%) than for patients aged 20–39 years (36%). Entry rates were higher among BTS patients (68%) than those treated elsewhere (43%).

Three successive trials for ES were open during the study period. The first of these was the UKCCSG study ET-1, which actually began in 1978 (Craft *et al*, 1997). The entry rate during 1980–1986 was 27% overall, 43% for children aged under 15 years and 19% for adolescents aged 15–19 years. The upper age limit for the study was 40 years, but only 4% of patients aged 20–29 years and none aged 30–39 were entered. Among children and adolescents, entry rates were much higher at (non-BTS) UKCCSG centres (59%) than elsewhere (14%).

The second ES trial was run jointly by UKCCSG and MRC (Craft *et al*, 1998). The entry rate overall for 1987–1992 almost doubled to 52%. Entry increased in all age groups below 30 years, reaching 72% for children under 15, 56% for adolescents aged 15–19 years and 33% for young adults aged 20–29 years. The upper age limit was 30 years and there were no entries above that age. Entry rates from UKCCSG and BTS centres (73%) were higher than elsewhere (19%).

The third trial, EICESS-92, was an international collaboration between UKCCSG, MRC and the German Co-operative Ewing's Sarcoma Study Group (Schuck *et al*, 2003). The upper age limit was 40 years. During 1993–1994, the first 2 years that this trial was open, the overall entry rate rose to 62%. This was largely due to an increase in the entry rate for children to 83%. Entry rates were 84% at UKCCSG and BTS centres and 15% elsewhere.

### Survival

**Figure 1:**
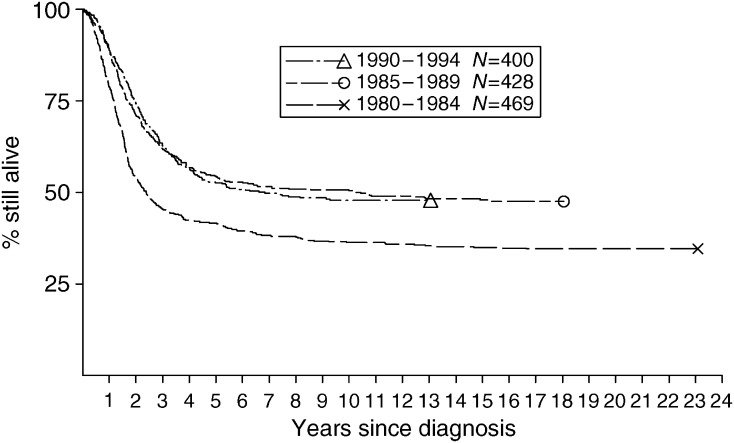
Survival of patients with OS diagnosed at age under 40 years during 1980–1994, by calendar period of diagnosis.

Table 2 shows the results of univariate analysis of survival by age, sex, primary site and year of diagnosis. Table 3 shows 5 year survival by period of diagnosis and age group. For OS diagnosed during 1990–1994, children aged under 10 years had a somewhat higher survival rate than older patients. Female subjects had a higher survival rate than males (Table 2). There was no evidence of a difference in survival between the sexes among children aged 0–14 years but female subjects had markedly higher survival in the older age groups. The variation in survival with age was present for both sexes, although less marked for female subjects (results not shown). Patients with skull tumours had an exceptionally high survival rate. Those with primaries in other axial sites (spine, ribs and sternum, pelvis) or in the arm had lower than average survival. Five-year survival rates during 1980–1984, 1985–1989 and 1990–1994 were 42% (95% CI: 37, 46), 54% (95% CI: 50, 59) and 53% (95% CI: 48, 57), respectively (Figure 1). The pattern of substantial increase in survival between 1980–1984 and 1985–1989 but no further improvement in 1990–1994 was similar for all age groups above age 10 years.

**Figure 2:**
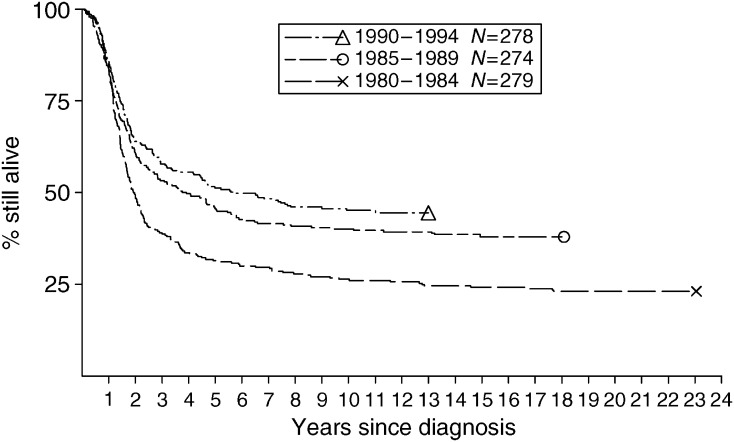
Survival of patients with ES diagnosed at age under 40 years during 1980–1994, by calendar period of diagnosis.

For ES, children aged under 15 years had higher survival than other patients. There was no difference in survival between male and female subjects. Pelvic primary site was associated with poor survival. The small number of patients with skull or hand primaries had a good prognosis. Survival for other axial sites was similar to that for limb primaries. Five-year survival rates during 1980–1984, 1985–1989 and 1990–1994 were 31% (95% CI: 26, 37), 46% (95% CI: 40, 51) and 51% (95% CI: 45, 57), respectively (Figure 2). This pattern of steadily increasing survival throughout the study period was present in each age group below 20 years, but above that age there was rather little change with calendar period.

Table 4 shows 5-year survival for patients at different categories of main treatment centre by period of diagnosis. For OS, survival during 1980–1984 was higher at BTS and UKCCSG centres than elsewhere. In 1985–1989, only the nonteaching hospitals had lower survival and in 1990–1994 there was relatively little variation.

For ES in all three calendar periods, survival was lower among patients treated at nonteaching hospitals. During 1985–1994, BTS and UKCCSG centres had higher survival rates than other teaching hospitals. Apart from the very few children aged under 10 years, patients of all ages at nonteaching hospitals had a lower survival rate. The higher survival at BTS centres was most marked for the age group 10–14 years.

Five-year survival of patients with OS who had surgery at a BTS centre in 1982–1984 was 59%, significantly higher than for those having surgery elsewhere (39%, log rank *P*=0.001). More recently, there was no significant difference in survival between BTS and other surgical centres (1985–1989, 58 *vs* 51%, *P*=0.26; 1990–1994, 51 *vs* 55%, *P*=0.85).

For OS, there was no significant difference in survival between trial and nontrial patients diagnosed during 1983–1986 (5-year survival 52 and 50%, respectively, log rank *P*=0.21) or 1987–1992 (5-year survival 50 and 55%, *P*=0.45).

**Figure 3:**
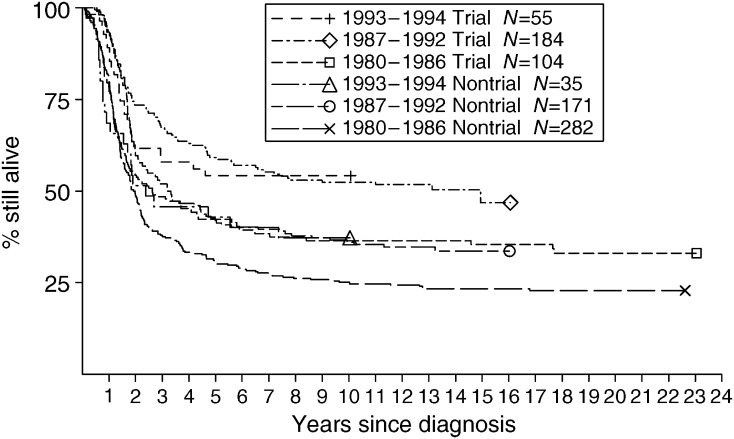
Survival of patients with ES diagnosed at age under 40 years during 1980–1994, by calendar period of diagnosis and trial status. The periods 1980–1986, 1987–1992 and 1993–1994 correspond, respectively, to periods of entry to the first UKCCSG study, the second UKCCSG-MRC study and the international trial EICESS-92.

Table 5 shows 5-year survival of trial and nontrial patients with ES. Year of diagnosis was regrouped as 1980–1986, 1987–1992, 1993–1994, to correspond with the periods when successive trials were open. Survival was higher for trial than for nontrial patients during the eras of the three successive trials (Figure 3). For patients aged under 20 years, survival was higher among those in the trials, but above that age there was no difference in survival between trial and nontrial patients.

In the univariate analysis of OS, the effects of main treatment centre and surgical centre differed for patients diagnosed before and after the end of 1984. Multivariate analysis was therefore performed for patients diagnosed during 1985–1994. The variables in the model were sex, age, primary site, main treatment centre and surgical centre. Results are shown in Table 6. Male sex and primary site in the spine, ribs and sternum or in the pelvis were confirmed as adverse prognostic factors. Compared with nonteaching hospitals as reference category of main treatment centre, survival of patients at UKCCSG and other non-BTS teaching hospitals was significantly higher.

For ES also, the effect of main treatment centre differed between 1980–1984 and 1985–1994, and multivariate analysis was therefore restricted to the latter period. Trial status was included in the model as it was significant in univariate analysis. Table 7 shows the results of two analyses, the first for all patients and the second only for those with a limb primary; surgical centre was included in the second analysis. Age 10–24 years and pelvic primary site were confirmed as adverse prognostic factors. Survival was significantly higher at BTS, UKCCSG and teaching hospital main treatment centres than at nonteaching hospitals. In a similar model in which treatment centre caseload was substituted for type of main treatment centre, those with fewer than two study patients per year had a significantly lower survival rate. In the analysis of patients with limb primaries, the adverse effect of age 10–24 years remained. Main treatment centre, surgical centre and trial status were all nonsignificant.

## DISCUSSION

Survival from both OS and ES increased markedly between 1980–1984 and 1985–1989. In the next 5 years, survival increased further for patients diagnosed with ES before the age of 20 years, although survival of those diagnosed at age 20–39 years fell back to near the level for 1980–1984. Among patients with OS diagnosed during 1990–1994, by contrast, only those aged under 10 years experienced any additional increase in survival compared with 1980–1989. Overall, these results are consistent with the contemporaneous decrease in population mortality from bone cancer, which was more marked at younger ages.

The favourable prognosis for craniofacial OS is in agreement with clinical reports (Gadwal *et al*, 2001; Smith *et al*, 2003). Data from the SEER Program cancer registries in the US showed no major difference compared with long bone primaries in an analysis including patients of all ages, although this did not allow for age, but the poor prognosis for pelvic OS was confirmed (Dorfman and Czerniak, 1995). In a more recent study of patients aged under 20 years, survival did not differ substantially by site, but no details were given (Gurney *et al*, 1999). In a recent clinical series, 40% of patients aged under 40 years with a pelvic OS had an inoperable primary or distant metastases (Grimer *et al*, 1999).

Among patients aged under 20 years with OS, we found that survival of those aged 10–14 years was lower than for younger children but there was no further deterioration of prognosis in the 15–19 year age group. In the USA, by contrast, survival was worse at age 15–19 than at age 10–14 years and children aged under 10 years at diagnosis also had a somewhat lower survival rate, particularly before 1985 (Gurney *et al*, 1999). The effects of age on survival in clinical series have been similarly inconsistent (Nagarajan *et al*, 2003).

Survival from OS was significantly higher for female than for male subjects. This is consistent with the higher metastasis-free survival for female subjects in the Scandinavian Sarcoma Group studies, in which it was suggested that that may be due to some unknown gender-dependent genetic factor (Smeland *et al*, 2003).

We found no difference in survival from ES between male and female subjects, whereas in the SEER data 5-year relative survival rates were 35% for males and 52% for female subjects (all ages, 1973–1987) and 50% for males, 68% for female subjects (age 0–19 years, 1975–1994). Survival rates from ES in the US have not been published for all ages, but among patients aged under 20 years pelvic primary site was associated with a low 5-year survival of under 35% (Gurney *et al*, 1999). The poor prognosis for pelvic tumours was also found in a large, combined clinical trial series from the UK and Germany and may be attributable to the larger tumour volume typical of that site (Cotterill *et al*, 2000).

A striking feature of the present study was the relation of survival to age for ES. The prognosis at age 15 years and over was much worse than for children below that age. In the UK – Germany clinical trial series, the age-related difference in survival was most marked with a cutoff point of 15 years (Cotterill *et al*, 2000). In the US, survival at age 10–14 years was lower than that for younger children, but adolescents aged 15–19 years had very similar survival to the 10–14 years age group (Gurney *et al*, 1999). In a study of patients with ES or pPNET treated on the adult sarcoma unit of the Royal Marsden Hospital, survival was higher for patients aged 20 years and over than for those aged 15–19 years (Verrill *et al*, 1997). That study only included 59 patients overall, and 51 aged 15–39 years. Moreover, patients with extra-osseous primaries were also included, although tissue of origin was not found to be of prognostic importance. In the present much larger study, there was a suggestion of increasing survival above age 20 years but this fell far short of statistical significance.

Survival of children aged under 15 years with OS during 1977–1980 did not vary between paediatric oncology centres and other hospitals, whereas in 1981–1984 there was a substantial increase in survival at paediatric oncology centres which did not take place elsewhere (Stiller, 1988). In the present study, covering 1980–1994, there was some indication that survival was higher at specialist centres than at other hospitals, but the differences were not always statistically significant. This is similar to the finding of nonsignificantly higher survival at major centres by Gill *et al* (1988), although their study largely related to an earlier period when modern combination chemotherapy for OS was not widely used and survival was much lower. During the 10 years for which data were available from national trials, slightly under half of the patients with OS in this study were entered in a trial. Survival was very similar for trial and nontrial patients. To be eligible for inclusion in the trials, patients had to have operable, high-grade OS and not to have other medical conditions which might preclude them from receiving treatment according to the protocol, and the maximum interval from biopsy to randomisation was 35 days (Bramwell *et al*, 1992; Souhami *et al*, 1997; Voûte *et al*, 1999). Most of these criteria would tend to exclude patients with a worse prognosis, although the histological criterion would exclude lower grade tumours with a better prognosis. There has been little progress in treatment and survival for OS since the early 1980s. It seems likely that trial and nontrial patients would have been similarly treated in the same hospitals and it is therefore not surprising that they should have similar survival rates. In the US Multi-Institutional Osteosarcoma Study, survival was similar for randomised patients and those who declined randomisation but were treated according to protocol (Link *et al*, 1991). The higher survival rate for patients who had surgery at a supraregional bone tumour centre was confined to the early years of the study, with little sign of any variation more recently. Within the European Intergroup trials during 1983–1993, survival rates were identical for patients treated at each of the three largest surgical centres despite different treatment policies (Grimer *et al*, 2002).

Children aged under 15 years with ES had a higher survival rate at paediatric oncology centres compared with other hospitals throughout the period 1977–1984 (Stiller, 1988). In the present study, survival was higher at specialist bone tumour treatment centres and paediatric oncology centres than at other hospitals, including other teaching hospitals. Survival of children and adolescents from ES was consistently higher throughout the study period for patients entered in national trials than for those who were not entered. Tumour stage was not available for this study and it is theoretically possible that any apparent effects of treatment centre and trial status on survival were due to confounding of these variables with stage. However, as primary site was an important prognostic factor associated with tumour burden, and trials were open for patients with both localised and metastatic disease, this seems unlikely. There was a maximum interval of 3 weeks between diagnosis and starting treatment for patients to be eligible for EICESS-92 (Paulussen *et al*, 2001), but no maximum period between diagnosis and starting treatment was specified for the two earlier trials (Craft *et al*, 1997; Craft *et al*, 1998). Patients with any primary site and those with metastatic disease at diagnosis were eligible for all three trials.

Our study has several strengths. We analysed a large series of patients from population-based cancer registries that would not be subject to the selection bias that can affect studies based on hospital data. This was a national study with the ability to describe and analyse patterns of care and survival for the whole country. While it might be possible to obtain more detailed data in a study of a smaller geographical area, such an area might have atypical patterns of care. Follow-up through national record systems was virtually complete. Information on place of treatment was available for the great majority of patients and trial status was determined by direct linkage with clinical trial databases.

The study also has a number of limitations. Information on stage of disease was not available and therefore we could not identify patients with an obviously poor outlook at diagnosis who may not have been referred to a tertiary centre or entered in a trial for that reason. If stage had been available, this information would not itself have been completely reliable as it need not have been based on identical investigations in every case. We were able, however, to make some allowance for extent of disease by using information on primary site, which is associated with tumour burden in both OS and ES. The effects of trial status and treatment centre in multivariate survival analyses which also included primary site were broadly similar to those in the univariate analyses. The most recent patients in the study were diagnosed just over 10 years ago. Meanwhile, entry rates to clinical trials could have increased, referral patterns could have changed and standards of treatment could have improved, especially in nonteaching hospitals. Survival studies are necessarily historical, however, and the results of clinical trials that were open at the end of our study period are yet to be published. Only a further study could determine whether patterns of care have changed more recently and, if so, how they have influenced survival.

In conclusion, within the limitations of an analysis of historic data with incomplete allowance for confounding factors, this study provides evidence of higher survival rates at tertiary centres for patients with both of the main types of bone sarcoma diagnosed at ages up to 40 years.

## Figures and Tables

**Table 1:** Main treatment centre for patients with osteosarcoma and Ewing's sarcoma

	**BTS**	**UKCCSG**	**Other teaching**	**Nonteaching**	**Unknown**	**Total**
(i) Treatment centre classified by type (see text)						
*Osteosarcoma*						
Age (years)						
0–9	14	76	9	10	1	110
10–14	110	162	37	27	4	340
15–19	127	52	184	61	42	466
20–39	119	0	205	64	45	433
						
1980–1984	70	88	173	105	45	481
1985–1989	151	89	150	38	26	454
1990–1994	149	113	112	19	21	414
						
Total	370	290	435	162	92	1349
						
*Ewing's sarcoma*						
Age (years)						
0–9	12	112	8	4	0	136
10–14	36	164	25	19	0	244
15–19	50	39	89	28	18	224
20–39	46	0	137	40	22	245
						
1980–1984	12	100	105	54	12	283
1985–1989	50	110	88	18	18	284
1990–1994	82	105	66	19	10	282
						
Total	144	315	259	91	40	849
						

(ii) Treatment centre classified by annual number of study patients (osteosarcoma and Ewing's sarcoma combined) referred per year						
	**⩾10 (BTS)**	**5–9**	**2–4**	**0–1**	**Unknown**	**Total**
*Osteosarcoma*						
Age (years)						
0–9	14	19	46	30	1	110
10–14	110	65	97	64	4	340
15–19	127	85	128	84	42	466
20–39	119	60	115	94	45	433
						
1980–1984	70	83	128	155	45	481
1985–1989	151	73	126	78	26	454
1990–1994	149	73	132	39	21	414
						
Total	370	229	386	272	92	1349
						
*Ewing's sarcoma*						
Age (years)						
0–9	12	21	76	27	0	136
10–14	36	49	97	62	0	244
15–19	50	62	56	38	18	224
20–39	46	56	58	63	22	245
						
1980–1984	12	62	114	83	12	283
1985–1989	50	74	86	56	18	284
1990–1994	82	52	87	51	10	282
						
Total	144	188	287	190	40	849

**Table 2:** Univariate analysis of survival by age, sex, primary site and year of diagnosis

	**Osteosarcoma**					**Ewing's sarcoma**				
	***N***	**1 year**	**5 years**	**10 years**	***P***	***N***	**1 year**	**5 years**	**10 years**	***P***
*Age (years)*										
0–4	9	67	67	56	0.39	27	85	52	48	<0.0001
5–9	99	91	58	55		109	89	59	56	
10–14	340	86	45	43		242	91	48	42	
15–19	446	85	47	41		216	81	30	26	
20–24	206	84	52	45		123	77	38	32	
25–29	82	79	48	45		62	82	40	32	
30–39	115	90	56	49		52	75	48	34	
										
*Sex*										
Male	759	85	47	41	0.031	485	83	43	37	0.61
Female	538	86	53	49		346	87	42	38	
										
*Primary site*										
Skull	52	94	67	67	<0.0001	25	92	56	56	<0.0001
Spine	21	76	43	29		58	79	43	36	
Ribs, sternum	21	67	38	33		88	86	45	38	
Pelvis	55	64	24	21		206	77	24	21	
Arm	157	86	44	35		102	88	53	47	
Hand						11	100	73	64	
Leg	964	87	51	47		284	90	49	42	
Foot	11	73	64	55		24	88	46	42	
Unspecified	16	69	50	44		33	64	39	36	
										
*Year of diagnosis*										
1980–1984	469	79	42	36	<0.0001	279	84	31	26	<0.0001
1985–1989	428	89	54	50		274	84	46	40	
1990–1994	400	89	53	48		278	86	51	45	

Percentage survival rates at 1, 5 and 10 years after diagnosis and *P*-value from log rank test for heterogeneity.

**Table 3:** Five-year survival (%) by calendar period and age at diagnosis

	**Osteosarcoma**				**Ewing's sarcoma**			
	**Age at diagnosis (years)**							
	**0–9**	**10–14**	**15–19**	**20–39**	**0–9**	**10–14**	**15–19**	**20–39**
*Year of diagnosis*								
1980–1984	45	39	41	45	40	37	15	35
1985–1989	54	51	54	57	67	49	26	47
1990–1994	71	48	48	55	72	59	47	39

**Table 4:** Five-year survival (%) for patients at different categories of main treatment centre by calendar period, 1980–1994

**Year of diagnosis**	***N***	**BTS**	**UKCCSG**	**Other teaching**	**Nonteaching**	**Unknown**	***P***
*Osteosarcoma*							
1980–1984	469	50	51	38	37	34	0.0092
1985–1989	428	54	58	54	37	76	0.077
1990–1994	400	49	55	55	44	56	0.49
							
*Ewing's sarcoma*							
1980–1984	279	33	40	31	21	8	0.0003
1985–1989	274	57	52	36	22	47	0.0001
1990–1994	278	57	59	42	11	56	<0.0001

**Table 5:** Five-year survival (%) for Ewing's sarcoma trial and nontrial patients by calendar period and age at diagnosis, 1980–1994

	***N***	**Trial**	**Non-trial**	***P***
*Year of diagnosis*				
1980–1986	386	42	30	0.0047
1987–1992	355	59	42	0.0001
1993–1994	90	54	43	0.093
				
*Age (years)*				
0–9	136	68	44	0.0005
10–14	242	52	40	0.024
15–19	216	48	19	<0.0001
20–24	123	38	38	0.83
25–39	114	43	44	0.72

**Table 6:** Results of Cox proportional hazards analysis for osteosarcoma diagnosed during 1985–1994

	**HR (95% CI)**
*Sex*	
Male	1 (reference)
Female	0.72 (0.59, 0.88)
	
*Age (years)*	
0–9	1 (reference)
10–14	1.30 (0.87, 1.94)
15–19	1.13 (0.74, 1.72)
20–39	1.06 (0.68, 1.63)
	
*Primary site*	
Skull	0.76 (0.43, 1.37)
Spine, ribs, sternum	1.84 (1.10, 3.10)
Pelvis	3.04 (2.07, 4.46)
Upper limb	1.10 (0.82, 1.47)
Lower limb	1 (reference)
Unspecified	2.34 (1.13, 4.84)
	
*Main treatment centre*	
BTS	0.86 (0.56, 1.32)
UKCCSG	0.60 (0.39, 0.93)
Teaching	0.69 (0.47, 1.00)
Nonteaching	1 (reference)
Unknown	0.42 (0.23, 0.75)
	
*Surgical centre*	
BTS	1 (reference)
Other	1.15 (0.88, 1.52)

HR=Hazard ratio.

**Table 7:** Results of Cox proportional hazards analysis for Ewing's sarcoma diagnosed during 1985–1994

	**HR (95%CI)**	
	**(i) All primary sites**	**(ii) Limb primaries only**
*Sex*		
Male	1 (reference)	1 (reference)
Female	0.94 (0.75, 1.18)	0.89 (0.63, 1.27)
		
*Age (years)*		
0–9	1 (reference)	1 (reference)
10–14	1.61 (1.04, 2.50)	2.05 (1.06, 3.97)
15–19	2.04 (1.27, 3.28)	3.08 (1.50, 6.31)
20–24	2.09 (1.21, 3.61)	3.18 (1.42, 7.10)
25–39	1.63 (0.94, 2.82)	1.95 (0.82, 4.61)
		
*Primary site*		
Skull	0.46 (0.19, 1.13)	—
Spine, ribs, sternum	1.20 (0.87, 1.66)	—
Pelvis	1.64 (1.24, 2.18)	—
Upper limb	0.87 (0.59, 1.28)	0.89 (0.61, 1.31)
Lower limb	1 (reference)	1 (reference)
Unspecified	1.18 (0.69, 2.00)	—
		
*Main treatment centre*		
BTS	0.44 (0.27, 0.71)	0.54 (0.24, 1.20)
UKCCSG	0.50 (0.30, 0.84)	0.57 (0.24, 1.32)
Teaching	0.64 (0.43, 0.97)	0.58 (0.29, 1.15)
Non-teaching	1 (reference)	1 (reference)
Unknown	0.42 (0.22, 0.80)	0.84 (0.29, 2.48)
		
*Surgical centre*		
BTS	—	1 (reference)
Other	—	1.32 (0.82, 2.12)
		
*Trial status*		
Trial	1 (reference)	1 (reference)
Nontrial	1.28 (0.97, 1.68)	1.31 (0.87, 1.97)
